# Metabolites of Pinang Yaki *(Areca vestiaria)* Fruit Extract: A Metabolite Profiling Study

**DOI:** 10.12688/f1000research.73758.3

**Published:** 2022-10-20

**Authors:** Herny Emma Inonta Simbala, Fahrul Nurkolis, Nelly Mayulu, Linda Wilhelma Ancella Rotty

**Affiliations:** 1 Pharmacy, Sam Ratulangi University, Manado, North Sulawesi, 95115, Indonesia; 2 Biological Sciences, State Islamic University of Sunan Kalijaga (UIN Sunan Kalijaga Yogyakarta), Yogyakarta, Yogyakarta, 55281, Indonesia; 3 Food and Nutrition, Sam Ratulangi University, Manado, North Sulawesi, 95115, Indonesia; 4 Internal Medicine, Sam Ratulangi University, Manado, North Sulawesi, 95115, Indonesia

**Keywords:** Mass spectrometry-based metabolomics, natural-based antivirus, pinang yaki, Areca vestiaria, SARS-CoV-2, medicinal plants, functional food

## Abstract

**Background:** Pinang yaki has bioactive compounds that have potential as a new herbal supplement. A better understanding of the bioactive compounds of pinang yaki using untargeted metabolomic profiling studies will provide clearer insight into the health benefits of pinang yaki and in particular its potential for the therapy and prevention of Covid-19.

**Methods:** Fresh samples of pinang yaki ( *Areca vestiaria*) are obtained from forests in North Sulawesi Province, Indonesia. Samples were used for untargeted metabolomics analysis by UPLC-MS.

**Results: **Based on an untargeted metabolomic profiling study of pinang yaki, 2504 compounds in ESI− and 2645 compounds in ESI+ were successfully obtained. After the analysis, 356 compounds in ESI− and 543 compounds in ESI+ were identified successfully. Major compounds Alpha-Chlorohydrin (PubChem ID: 7290) and Tagatose (PubChem ID: 439312) were found in ESI+ and ESI−.

**Discussion: **The Top 10 metabolites from pinang yaki extract (ESI+) juga have been indicated in preventing SARS Cov2 infection and have exhibited good neuroprotective immunity. Benzothiazole (PubChem ID: 7222), L-isoleucine (PubChem ID: 6306), D-glucono-delta-lactone (PubChem ID: 736), Diethylpyrocarbonate (PubChem ID: 3051), Bis(2-Ethylhexyl) amine (PubChem ID: 7791), Cinnamic acid (PubChem ID: 444539), and Trigonelline (PubChem ID: 5570) also had potential effects as an antiviral, anti-inflammatory, and anti-Covid19.

**Conclusion: **Untargeted metabolomic profiling showed many bioactive compounds contained in pinang yaki ( *Areca vestiaria*) extract. The top 10 compounds have been identified and explored for their potential benefits as anti-Covid19 supplement products. This is a preliminary study which still needs further research such as preclinical and clinical trials.

## Introduction

Coronavirus disease 19 (COVID-19) is a highly communicable and deadly virus caused by the severe acute respiratory syndrome coronavirus 2 (SARS-CoV-2) that first appeared in Wuhan, China in December 2019 and has since spread around the world ( Mouffouk et al., 2021). The SARS-CoV-2 virus is a β-coronavirus, a non-segmented enveloped positive-sense RNA virus with a crown-like appearance and symmetric helical nucleocapsid ( Astuti & Ysrafil, 2020). Unlike the previous outbreaks of coronavirus, SARS-CoV-2 is more transmittable and the majority of those infected remain asymptomatic, resulting in ineffective containment and mitigation ( Andersen et al., 2020). It has become a major cause of mortality and morbidity worldwide ( Das et al., 2021). Therefore, finding an optimal therapeutical solution is vital.

Medicinal plants and natural products can be promising alternatives to supplements or drugs to treat and prevent diseases ( Benarba & Pandiella, 2020). Pinang yaki *(Areca vestiaria)*, also known as crown shaft palm, is an endemic palm plant that grows in North Sulawesi that is traditionally utilized for the treatment of diabetes and diarrhoea, and as a contraceptive ( Gosal et al., 2017; Herny et al., 2010). Pinang yaki fruit extract has bioactive compounds such as flavonoids, tannins, saponins, triterpenoids, and hydroquinones, which are known primarily as natural antioxidants ( Simbala et al., 2017). Tannins and flavonoids are known for their antitumor, antiallergic, antihepatotoxic, and antioxidant activities ( Londok et al., 2017), while triterpenoids exert antibacterial, anticancer, anti-inflammation, and wound care properties ( Herny et al., 2010), as well as inhibiting viral replication ( Das et al., 2021). Saponins also show antifungal, cytotoxic, antibacterial, and antiviral properties ( Kregiel et al., 2017). A recent review also stated that flavonoids may inhibit viral replication and translation ( Ahmad et al., 2015), enhance immune activity, antiviral protection, and reduce respiratory problems ( Yang et al., 2020); since phenols inhibit the fusion of the virus to the host cell ( Das et al., 2021). Further separation and analysis of pinang yaki fruit extract also identified pentadecane and hexadecanoic acid ( Simbala et al., 2017), which were known for their ability in downregulating pro-inflammatory cytokines ( Chuah et al., 2018; Aparna et al., 2012). Experimental animal model also revealed lipid-lowering properties of ethanol extract of pinang yaki fruit ( Sagay, Simbala, & De Queljoe, 2019).

Despite all those health-beneficial properties, pinang yaki is still underutilized. Pinang yaki and its bioactive compounds have the potential as a novel herbal supplement. A better understanding of pinang yaki’s bioactive compounds using an untargeted metabolomic profiling study will provide clearer insight into the health benefits of pinang yaki, in particular its potential for therapy and prevention of viral infection. It is hoped that through this study, the metabolites that may play important roles in sustaining life and health can be explored as stated in the article of Vinayavekhin and Saghatelian (2010) and Vinayavekhin (2013). More specifically, the possible potential of the metabolites of areca yaki as antiviral.

## Methods

Fresh samples of pinang yaki ( *Areca vestiaria*) fruit with the age of 3.5 months were obtained from Bolaang Mongondow forest in North Sulawesi Province, Indonesia. The samples were then cleaned using distilled water (Aquades) and then stored in a cooling box to be sent to the intended metabolomic testing laboratory. Samples were used for untargeted metabolomics analysis by UHPLC-MS. The botanical identification and authentication were confirmed at the Department of Pharmacology, Faculty of Mathematics and Natural Sciences, Sam Ratulangi University, Indonesia by comparing the specimen with previously identified reference specimens ( Applequist & Miller, 2013). Untargeted metabolomics analysis and identification of compounds were conducted at *Apical Scientific Sdn. Bhd.* 43300 Seri Kembangan laboratory, Selangor, Malaysia with registration number #CPMO08102001a.

### Instruments and reagents

Ultimate 3000LC combined with Q Exactive MS (Thermo Fisher), Temp functional Centrifugation ( Eppendorf), ACQUITY UHPLC HSS T3 (100 × 2.1 mm × 1.8 μm), Acetonitrile ( Merck), Methanol ( Merck), Formic acid (CNW).

### Sample preparation

Samples were thawed, and 50 mg of each sample was precisely weighed into a tube, add with 800 μL of 80% methanol with vortex for 90 s, and followed by sonication for 30 min, 4 °C. Then all samples were kept at −40 °C for 1 h. After that, samples were vortexed for 30 s, kept for 0.5 h, and centrifuged at 12000 rpm and 4 °C for 15 mins. Finally, 200 μL of supernatant was transferred to a vial for LC-MS analysis.

### Liquid chromatography-mass spectrometry (LC-MS)

Performed by Ultimate 3000LC combined with Q Exactive MS (Thermo Fisher) and screened with electrospray ionization-mass spectrometry (ESI-MS). The LC system is comprised of an ACQUITY UHPLC HSS T3 (100 × 2.1 mm, 1.8 μm) with Ultimate 3000LC. The mobile phase is composed of solvent A (0.05% formic acid-water) and solvent B (acetonitrile) with a gradient elution (0-1.0 min, 95% A; 1.0-12.0 min, 95-5% A; 12.0-13.5 min, 5% A; 13.5-13.6 min, 5-95% A; 13.6-16.0 min, 95% A). The flow rate of the mobile phase is 0.3 mL·min ^−1^. The column temperature is maintained at 40 °C, and the sample manager temperature is set at 4 °C. A 40 μL injection of metabolite extract was made into the system for every run.

Mass spectrometry parameters in positive ion mode (ESI+) and negative ion mode (ESI−) mode are listed as follows:

• ESI+: Heater Temp 300 °C; Sheath Gas Flow rate, 45arb; Aux Gas Flow Rate, 15arb; Sweep Gas Flow Rate, 1arb; spray voltage, 3.0 KV; Capillary Temp, 350 °C; S -Lens RF Level, 30%.
• ESI−: Heater Temp 300 °C, Sheath Gas Flow rate, 45arb; Aux Gas Flow Rate, 15arb; Sweep Gas Flow Rate, 1arb; spray voltage, 3.2 KV; Capillary Temp, 350 °C; S -Lens RF Level, 60%.

## Results

**Figure 1.**
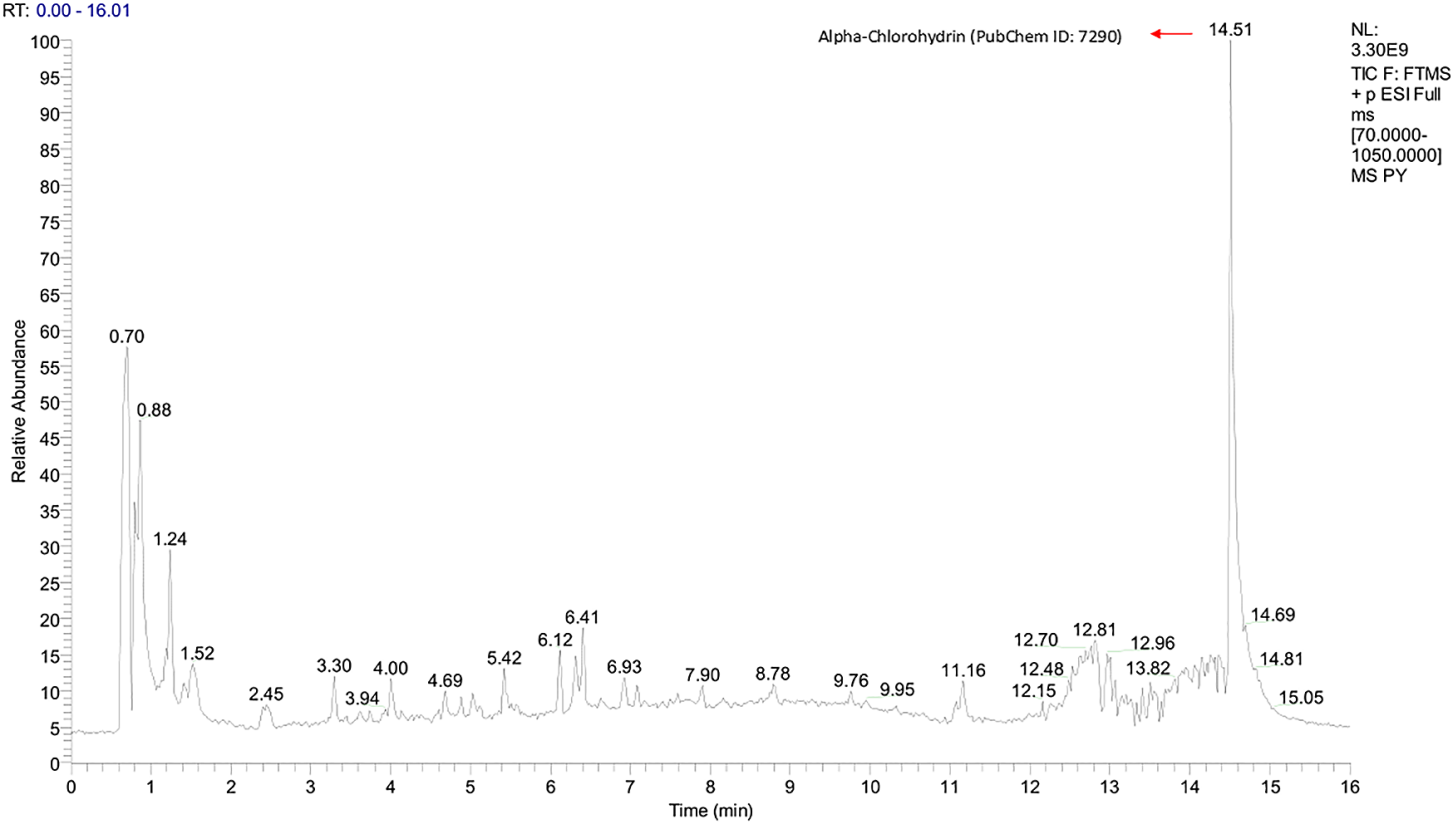
Total-Ion Current (TIC) was obtained from Pinang Yaki in ESI+ mode. Based on an untargeted metabolomic profiling study of pinang yaki, 2645 compounds in ESI+ were successfully obtained ( Nurkolis & Simbala, 2021). After the analysis, 543 compounds in ESI+ were successfully identified ( Nurkolis & Simbala, 2021). Figure 1 shows that the major compound was Alpha-chlorohydrin (PubChem ID: 7290).

**Figure 2.**
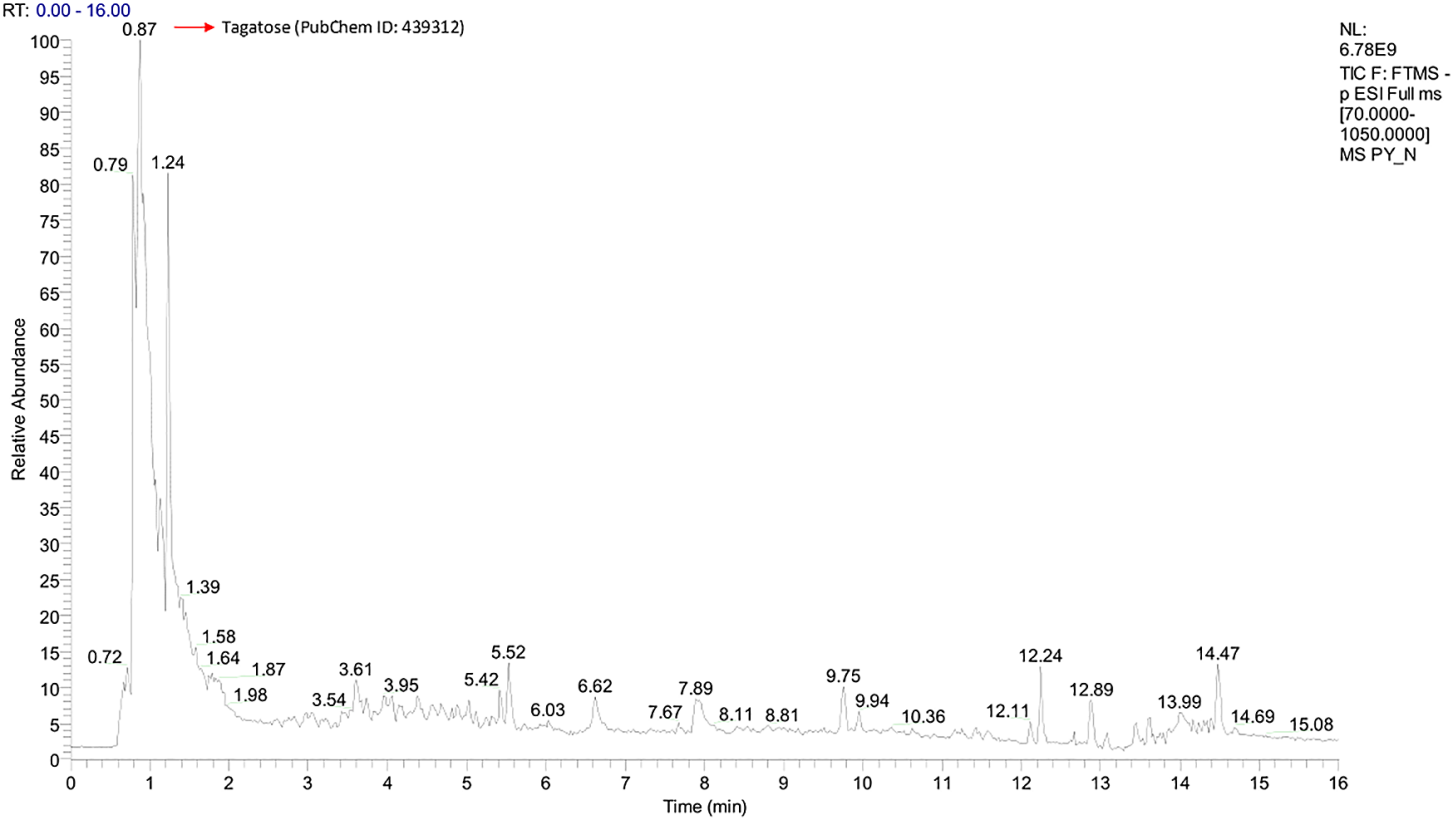
Total-Ion Current (TIC) was obtained from Pinang Yaki in ESI− mode. In ESI−, a total of 2504 compounds were obtained, and 356 compounds were successfully identified by name (see underlying) ( Nurkolis & Simbala, 2021). Figure 2 shows that the identified major compound was tagatose (PubChem ID: 439312).

Graphs shown in Figures 1 and 2 were used to determine the peak representing the number of annotated ions, retention time, and relative abundance of the ions. This data was then used to identify the compounds contained in the sample extract. The results of identification and mass of compounds were carried out by the laboratory of Apical Scientific Sdn. Bhd and the results provided in an Excel spreadsheet ( Microsoft Excel, RRID:SCR_016137) ( see data availibity statement) ( Nurkolis & Simbala, 2021). Based on an untargeted metabolomic profiling study of pinang yaki, 2504 compounds in ESI− and 2645 compounds in ESI+ were successfully obtained ( Nurkolis & Simbala, 2021). After the analysis, 356 compounds in ESI− and 543 compounds in ESI+ were identified successfully ( Nurkolis & Simbala, 2021). From each chromatogram, the strength of the various adducts was roughly measured.

After 543 compounds in ESI+ were successfully identified, those compounds were ranked into the top 10 metabolites present in pinang yaki extract (ESI+) which have best capability to ionize in positive modes, as is shown in Table 1.

**Table 1.** Top 10 metabolite from Pinang Yaki (ESI+).

Molecular weight (g/mol)	m/z	RT (min)	Name	Peak area	PubChem ID
103.09999	104.1073	0.818	Choline	24527.6	305
135.01414	136.0214	6.321	Benzothiazole	15776.47	7222
131.09455	132.1018	1.529	L-Isoleucine	15164.33	6306
110.01313	111.0204	14.528	Alpha-Chlorohydrin	14077.62	7290
178.04759	179.0549	0.876	D-Glucono-Delta-Lactone	12736.37	736
162.05267	163.0599	0.839	Diethylpyrocarbonate	10537.28	3051
241.27652	242.2838	6.909	Bis(2-ethylhexyl)amine	7771.887	7791
148.05225	149.0596	2.442	Cinnamic Acid	7221.059	444539
96.02104	138.0548	0.837	Trigonelline	6500.932	5570
184.00067	185.008	1.418	Chelidonic Acid	6135.752	7431

In Table 2, the ranking is also shown for ESI−, providing the top 10 metabolite compounds which have best capability to ionize in negative we have modes. Tables 1 and 2 show that chelidonic acid (PubChem ID: 7431) was present in both top 10 metabolites.

**Table 2.** Top 10 metabolite from Pinang Yaki (ESI−).

Molecular weight (g/mol)	m/z	RT (min)	Name	Peak area	PubChem ID
184.0002	182.9924	1.264	Chelidonic Acid	97389.49	7431
196.0573	195.05	0.799	Gluconic Acid	44676.74	10690
192.0261	191.0188	0.901	Citric Acid	21807.88	311
88.01464	87.0073	0.965	Pyruvic Acid	169834.1	1060
156.0181	155.0104	0.819	Propanediol 1-Phosphate	15028.76	440156
134.0201	133.0129	1.166	D-(+)-Malic Acid	14024.67	92824
166.9852	165.9779	6.659	2-Mercaptobenzothiazole	10616.37	697993
116.0096	115.0023	0.975	Maleic Acid	10462.28	444266
273.966	272.9587	0.698	1-O-Arsonopentofuranose	9882.362	25201247
180.0624	179.055	0.885	Tagatose	7052.94	439312

## Discussion

The richness of Indonesia's natural ingredients and their active compounds still needs to be revealed and explored, in connection with the discoveries of new natural-based drug candidates. This untargeted metabolomic profiling study was conducted to clarify the content of compounds contained in pinang yaki *(Areca vestiaria)* and to see its potential as a Covid-19 and antiviral herbal remedy by literature study. But of course, this study is a basic study that does not necessarily represent efficacy in animals (preclinical studies) and clinical trials in humans. However, the content of compounds that have been successfully identified in this study can be used as a basic reference in determining the dose of trials in animals (preclinical study).

Tables 1 and 2 show that chelidonic acid (PubChem ID: 7431) is one of metabolites identified to ionize well using either ESI technique. A study conducted by Shin et al. (2011), showed the inhibitory effects of chelidonic acid on IL-6 production by blocking NF-κB and caspase-1 in HMC-1 cells that can be a potential therapy for inflammatory diseases, including Covid-19 ( Shin et al., 2011; Zhang et al., 2020). In addition, Tagatose (PubChem ID: 439312) which occupies the highest peak of ESI− mode ( Figure 2), indicates that its presence in pinang Yaki *(Areca vestiaria)* is also quite high. Studies have shown that it can be a functional antidiabetic food for diabetes, which according to meta-analysis, is comorbid for Covid-19 patients ( Guerrero-Wyss et al., 2018; Kun’ain et al., 2020). Alpha-chlorohydrin, also topped the highest position in ESI+ mode ( Figure 1), which has a potential immunostaining effect in people with declining viral infections such as SARS Cov-2 ( Soliman et al., 2014). However, there are negative effects of the use of Alpha-chlorohydrin on epididymis rats, therefore a comprehensive follow-up study is needed to look at the beneficial effects as well as their toxicity ( Soliman et al., 2014).

In addition, choline (PubChem ID: 305) which is the metabolite from pinang yaki extract (ESI+) has also been widely researched for its effects in preventing SARS Cov-2 Infection and has good neuroprotective immunity ( Chowdhury & Pathak., 2020). Other contents in pinang yaki *(Areca vestiaria)* such as Benzothiazole (PubChem ID: 7222) ( Ali & Siddiqui., 2013), L-Isoleucine (PubChem ID: 6306) ( Mao et al., 2018), D-Glucono-Delta-Lactone (PubChem ID: 736) ( Kuwano et al., 2018), Diethylpyrocarbonate (PubChem ID: 3051) ( Yamamura & Cochran., 1976), Bis(2-ethylhexyl) amine (PubChem ID: 7791) ( Nazemi et al., 2014), Cinnamic acid (PubChem ID: 444539) ( Gravina et al., 2011), and Trigonelline (PubChem ID: 5570) (Zhou et al., 2021) also have potential effects as an antiviral, anti-inflammatory, and anti-Covid19 agents.

Additionally, in ESI− mode, some of the highest-grade compounds in pinang yaki extract, such as gluconic acid (PubChem ID: 10690), citric acid (PubChem ID: 311), pyruvic acid (PubChem ID: 1060), propaediol 1-phosphate (PubChem ID: 440156), D-(+)-malic acid (PubChem ID: 92824), 2-mercaptobenzothiazole (PubChem ID: 697993), maleic acid (PubChem ID: 444266), and 1-O-arsonopentofuranose (PubChem ID: 25201247) have anti-inflammatory and antiviral effects.

The above compounds have been identified as contained in pinang yaki extract, which shows its potential as an antiviral herbal supplement product. Of course, it needs further study, researchers will conduct an *in vitro*, *in vivo* or preclinical study to find out the effect of pinang yaki extract as an antiviral herbal supplement (based on the results of the compounds or metabolomic profiling in this paper). In addition, with reference to the metabolite data from this study, it is highly recommended that further studies be carried out in the future, namely the abundance of these metabolites in the sample and molecular mechanisms against several viral infection receptors through computational studies or in silico molecular docking with molecular dynamics simulations.

## Conclusion

This untargeted metabolomic profiling study shows many bioactive compounds are contained in pinang yaki *(Areca vestiaria)* extract. The top 10 compounds which can ionize well and their potential benefits as antiviral supplement products have been identified and explored by literature study. The study of *in vitro*, preclinical, and clinical trials of Pinang yaki metabolites with biological mechanism is urgently needed.

## Data availability

### Underlying data

**Figshare:** Pinang Yaki ( *Areca vestiaria*) Extract by Untargeted Metabolomic Profiling. https://doi.org/10.6084/m9.figshare.16560276.v1 ( Nurkolis & Simbala, 2021).

The project contains the following underlying data:

• Raw Data for Untargeted Metabolomic Profiling of Pinang Yaki *(Areca vestiaria)* Extract ESI+ and ESI−.

Data are available under the terms of the Creative Commons Zero "No rights reserved" data waiver (CC0 1.0 Public domain dedication).
