# Supplementary material for: Metabolites of Pinang Yaki (Areca vestiaria) Fruit Extract: A Metabolite Profiling Study
Source: F1000Res. 2022 Oct 20;10:1021. Originally published 2021 Oct 8. [Version 3] doi: 10.12688/f1000research.73758.3 (PMC10724647; doi:10.12688/f1000research.73758.3)
Supplement: Peer review history

# Reviewer report: Reviewer response for version 3

**DOI:** 10.5256/f1000research.139901.r226910

**Published:** 2023-12-15

**Authors:** Tooba Mahboob

**Affiliations:** 1 UCSI University, Kuala Lumpur, Malaysia

The manuscript by Simbala et al. explores the metabolites of Pinang yaki fruit extract for various biological activities. The authors hypothesise that identified compounds from Pinang yaki fruit extract has antiviral and neuroprotective potential which warrants further studies. The following issues need to be addressed by authors:

 1- Certain names such as COVID-19, SARS-CoV-2, should be capitalized throughout the manuscript. Terminologies like In silico etc should be italicized. Ensure the consistency in terms throughout the manuscript.

 2- Introduction. More relevant papers should be cited. Tannins and flavonoids are broader chemical class of compounds, isolated from Pinang yaki fruit. Please specify some of the names of these chemical compounds which have been identified in prior studies. Similarly in the next line, certain biological activities of saponins have included. It would be better to include the name of saponin.

 3- Methods: How was plant material cutting, pulverizing or drying done?. Need to include details in methods.

 Please state if triplicates were done for UHPLC-Ms studies.

Is the work clearly and accurately presented and does it cite the current literature?

Partly

If applicable, is the statistical analysis and its interpretation appropriate?

Not applicable

Are all the source data underlying the results available to ensure full reproducibility?

Yes

Is the study design appropriate and is the work technically sound?

Yes

Are the conclusions drawn adequately supported by the results?

Yes

Are sufficient details of methods and analysis provided to allow replication by others?

Yes

Reviewer Expertise:

Natural Products, Microbiology, Nanotechnology

I confirm that I have read this submission and believe that I have an appropriate level of expertise to confirm that it is of an acceptable scientific standard.

# Reviewer report: Reviewer response for version 2

**DOI:** 10.5256/f1000research.121189.r137719

**Published:** 2022-05-23

**Authors:** Nawaporn Vinayavekhin

**Affiliations:** 1 Center of Excellence in Natural Products Chemistry, Department of Chemistry, Faculty of Science, Chulalongkorn University, Bangkok, Thailand

In this manuscript, the authors identified metabolites in pinang yaki fruit extract using LC-MS and tried to link these metabolites to their anti-COVID19 properties. However, in my opinion, many points need to be improved before this manuscript can be indexed.

1. Title: The title does not represent accurately what the authors did. The word “untargeted metabolomics profiling” should be reserved for comparative studies. In addition, the authors did not perform experiments to show the relevance of these metabolites or even the pinang yaki itself on Covid-19 prevention or treatment at all, and thus, this should not be in the title. The appropriate title should be more like “Metabolite profiling of pinang yaki ( *Areca vestiaria*) fruit” or something similar.
2. Introduction: The authors just mentioned the classes of metabolites in pinang yaki fruit, such as flavonoids, tannins, saponins, etc. As these classes of metabolites are quite common in plants, the statement did not give much information. Are there any known specific bioactive metabolites in pinang yaki fruit? If so, this should be mentioned.
  Also, are there any known properties or benefits of pinang yaki fruit or extract at all?
3. The authors mentioned the use of UPLC-MS, but from the name of the instrument given, it’s more like UHPLC system, rather than UPLC.
4. How exactly were the botanical identification and authentication carried out?  
5. How many replicates of samples were carried out? I looked up the authors’ raw data on the Excel spreadsheet, and the headings of the two columns are BH and PY. It’s unclear what these abbreviations stand for. Are these the two replicates of the sample? In any case, more replicates are likely required to make sure that what the authors observed are not just some contaminants that got picked up by LC-MS.
6.  Injection volume for LC-MS analysis should be specified.
7. The details of the data analysis method are needed. In addition, as metabolite identification is the main content of this report, it should be discussed in detail and should be done rigorously. How exactly were metabolites identified, i.e. by accurate mass, MS/MS, the use of standard, or the combination thereof? With the identification of tagatose, for example, how can you know it’s not glucose or other sugars? From the structure, it should ionize better in the positive ion mode than in the negative ion mode as well, so it’s a surprise that the authors did not observe a higher peak of tagatose in the positive ion mode.
8. Do the numbers of compounds detected in the positive and negative ion modes include isotopes and various adducts? How could you distinguish these from real compounds?
9. It’s unclear how metabolites are ranked. Is it by peak area? However, the peak area in ESI-MS does not correlate with the abundance of metabolites, as different compounds have differing abilities to ionize in different modes. Thus, the levels of these different metabolites cannot be compared simply by peak areas. In addition, the statement that chelidonic acid could be found in the top 10 metabolites lists in both modes just means that it possesses the structure that allows it to ionize very well in both modes. It does not have any significance other than this.

If the authors want to really investigate the abundance of these metabolites in the sample, they will have to acquire the standard for each compound, and compare its abundance to the constructed standard curve.

Is the work clearly and accurately presented and does it cite the current literature?

Partly

If applicable, is the statistical analysis and its interpretation appropriate?

Not applicable

Are all the source data underlying the results available to ensure full reproducibility?

No source data required

Is the study design appropriate and is the work technically sound?

No

Are the conclusions drawn adequately supported by the results?

No

Are sufficient details of methods and analysis provided to allow replication by others?

No

Reviewer Expertise:

LC-MS-based metabolomics

I confirm that I have read this submission and believe that I have an appropriate level of expertise to state that I do not consider it to be of an acceptable scientific standard, for reasons outlined above.

# Reviewer report: Reviewer response for version 2

**DOI:** 10.5256/f1000research.121189.r123459

**Published:** 2022-02-28

**Authors:** Toto Sudargo

**Affiliations:** 1 Department of Nutrition and Health, Faculty of Medicine, Public Health, and Nursing, Universitas Gadjah Mada, Yogyakarta, Indonesia

I don't have any further comments.

Is the work clearly and accurately presented and does it cite the current literature?

Yes

If applicable, is the statistical analysis and its interpretation appropriate?

Yes

Are all the source data underlying the results available to ensure full reproducibility?

Yes

Is the study design appropriate and is the work technically sound?

Yes

Are the conclusions drawn adequately supported by the results?

Yes

Are sufficient details of methods and analysis provided to allow replication by others?

Yes

Reviewer Expertise:

Health and Nutrition

I confirm that I have read this submission and believe that I have an appropriate level of expertise to confirm that it is of an acceptable scientific standard.

# Reviewer report: Reviewer response for version 1

**DOI:** 10.5256/f1000research.77431.r118669

**Published:** 2022-01-31

**Authors:** Zanariah Hashim

**Affiliations:** 1 School of Chemical and Energy Engineering, Faculty of Engineering, Universiti Teknologi Malaysia, Skudai, Malaysia

1. Please check the grammar. Specific names require capitalization (for example, department of pharmacology, faculty of mathematics and natural sciences, should be changed to Department of Pharmacology, Faculty of Mathematics and Natural Sciences), whereas compound names do not need to be capitalized (e.g. Chelidonic Acid --> chelidonic acid).
2. Which part of the plant was used as the extract? How old is the plant? Include more specifically where the plant comes from (not just some forests in North Sulawesi).  Additionally, the authors need to explain the sample preparation procedure more clearly (including cutting, washing, sorting, etc.)
3. Explain how the compound identification was performed. This is the most vital part of this work.
4. Ref in Figure 1: please cite correctly: Nurkolis & Simbala.
5. What does "quantity" in Table 1 and Table 2 refer to? Is it peak area or peak height? Higher peak area/height does not necessarily mean that the compound is more abundant in the sample. It simply means the compound is easier (more sensitive) to be detected using the method. To confirm the abundance/concentration of the compound, the authors need to analyze standard compounds and draw calibration curves.
6. This is a preliminary result of untargeted metabolomic analysis of pinang yaki extract (which part should be mentioned clearly). Although the discovered compounds may have the potential to treat Covid-19, the discussion is based only on literature, without evidence from the authors themselves. As such, I think some parts are overly-discussed and the title is also misleading. Discussion can be improved by adding pathway analysis or other multivariate analysis.

Is the work clearly and accurately presented and does it cite the current literature?

Yes

If applicable, is the statistical analysis and its interpretation appropriate?

Partly

Are all the source data underlying the results available to ensure full reproducibility?

Yes

Is the study design appropriate and is the work technically sound?

Yes

Are the conclusions drawn adequately supported by the results?

Partly

Are sufficient details of methods and analysis provided to allow replication by others?

Partly

Reviewer Expertise:

metabolomics, LC-MS, multivariate analysis

I confirm that I have read this submission and believe that I have an appropriate level of expertise to confirm that it is of an acceptable scientific standard, however I have significant reservations, as outlined above.

# Reviewer report: Reviewer response for version 1

**DOI:** 10.5256/f1000research.77431.r96491

**Published:** 2021-10-29

**Authors:** Toto Sudargo

**Affiliations:** 1 Department of Nutrition and Health, Faculty of Medicine, Public Health, and Nursing, Universitas Gadjah Mada, Yogyakarta, Indonesia

1. The discussion should be more expressive. The contribution of this study to the literature should be highlighted.
2. Please ensure the manuscript is proofread and edited as there is a significant number of grammatical errors throughout the manuscript.
3. The abstract description is good, there is no need to rewrite to provide details. The most important result obtained from the study should be emphasized in this section.
4. Conclusion: Please rewrite. It should not be the summary of the findings as this has been stated in the Results. The conclusion should be more concise and match the aim.

It can be seen from the research, that this research gives us new insights into the world of science, especially on the problem of Covid-19.

Is the work clearly and accurately presented and does it cite the current literature?

Yes

If applicable, is the statistical analysis and its interpretation appropriate?

Yes

Are all the source data underlying the results available to ensure full reproducibility?

Yes

Is the study design appropriate and is the work technically sound?

Yes

Are the conclusions drawn adequately supported by the results?

Yes

Are sufficient details of methods and analysis provided to allow replication by others?

Yes

Reviewer Expertise:

Health and Nutrition

I confirm that I have read this submission and believe that I have an appropriate level of expertise to confirm that it is of an acceptable scientific standard.
